# Radical Prostatectomy as a First-Line Treatment in Patients with Initial PSA  >20 ng/mL

**DOI:** 10.1155/2012/832974

**Published:** 2012-07-19

**Authors:** Alexander I. Hinev, Deyan Anakievski, Vesselin I. Hadjiev

**Affiliations:** ^1^Clinic of Urology, Department of Surgery, “St. Marina” University Hospital, Hr. Smirnenski Street 1, 9010 Varna, Bulgaria; ^2^Department of Statistics, University of Economics, 9010 Varna, Bulgaria

## Abstract

Initial PSA >20 ng/mL is generally considered an adverse prognostic feature in prostate cancer (PCa). Our goals were to estimate the impact of radical prostatectomy (RP) on biochemical recurrence- (BCR-) free and cancer-specific survival (CSS) rates of PCa patients with PSA >20 ng/mL, and to identify patients with favorable oncological outcome. Using 20 ng/mL as a cut-point value, 205 PCa patients, who underwent RP, were stratified into two groups. Multivariate analysis was used to determine the significant outcome predictors among patients with PSA >20 ng/mL. Men in this group had significantly lower 10-yr BCR-free and CSS rates than patients with PSA ≤20 ng/mL (20.7% versus 79.6% (*P* < 0.001) and 65.0% versus 87.9% (*P* = 0.010), resp.). Pathological stage and lymph node status were found to be the only independent predictors of PSA failure. Patients with favorable combination of these variables (pT2, N0) had significantly longer 10-yr BCR-free and CSS rates (44.3% versus 0% (*P* = 0.001) and 100.0% versus 33.6% (*P* = 0.011), resp.). High PSA values do not uniformly indicate poor prognosis after surgery. Patients, who might benefit the most from RP, are those with organ confined PCa and negative lymph nodes.

## 1. Introduction

The stage migration of prostate cancer (PCa), due to its prostate-specific antigen (PSA-) based early detection, dramatically changed the pattern of presentation in many patients with potentially lethal disease. Nowadays, an increasing number of patients are initially diagnosed with cancer confined to the prostate. However, approximately one third of these men are found to have aggressive pathological features by the final histological report: extraprostatic extension (EPE), seminal vesicle invasion (SVI), and/or lymph node involvement (LNI) [1, 2]. These numbers could be even higher, if a more aggressive treatment policy of performing radical prostatectomy (RP) is implemented [3, 4].

 PSA is one of the most established tumor markers that is widely used in screening, diagnosis, staging, and monitoring of prostate cancer patients [5, 6]. PSA has an established prognostic impact and is one of the three basic parameters (together with the biopsy Gleason score and the clinical stage) that is included in all preoperative prognostic tools [5, 7–9].

 Serum PSA above 20 ng/mL is generally considered as an adverse prognostic feature in PCa, associated with a higher prevalence of a locally advanced disease and/or distant metastases [10, 11] and with a higher probability of developing recurrent disease after radical local treatment [7, 9, 12]. Therefore, many urologists are reluctant to perform RP on patients with PSA values >20 ng/mL [13–15].

 Some contemporary studies in which patients are diagnosed earlier suggest, however, that the risk may not be so dire [14, 16–21], as some patients, subjected to RP, showed favorable outcomes despite high PSA values [13, 18–23].

 In addition, adjuvant treatment has been used in such patients with contradictory results, with some studies suggesting that there is no benefit from adjuvant treatment, while many others claim the opposite [24–28].

 Therefore, two issues need more clarification: what is the exact detriment to having initial PSA values above 20 ng/mL, and whether adjuvant treatment may benefit this particular subset of patients.

 The main goals of the present study were: (1) to estimate the impact of radical prostatectomy on biochemical recurrence- (BCR-) free and cancer-specific survival (CSS) rates of patients with PCa and PSA >20 ng/mL and (2) to identify a subset of patients who might have a favorable oncological outcome.

## 2. Materials and Methods

 Since April 1996, a total of 205 male patients, aged between 46 and 79 years (mean age 65.6 ± 6.7 years), underwent extended pelvic lymph node dissection (ePLND), followed by RP for localized or locally advanced PCa (Table 1). Digital rectal examination (DRE) and transrectal ultrasound (TRUS) of the prostate were used as the compulsory initial staging procedures. They were supplemented by an abdominal and pelvic computer tomography (CT) or magnetic resonance imaging (MRI) and bone scintigraphy in case of a palpable bulky tumor of the prostate, initial PSA >20 ng/mL, or biopsy Gleason score ≥8. Patients with preoperatively proven metastatic disease were considered not eligible for radical surgery.

Seventy-one patients, included in the present study, had already received some form of neoadjuvant hormonal therapy (Table 2). Twelve of these patients had bilateral orchiectomy performed prior to surgery. The decision to start this type of therapy had been taken at the primary urological institution, where the disease had been detected. Interestingly, only 33 (46.5%) of these 71 patients had initial PSA >20 ng/mL, while 38 (53.5%) patients had initial PSA below this crucial cut-point value.

 The patients were informed in detail about the study objectives and the study protocol and about all potential side effects and complications that might be associated with it. All patients gave their written consent prior to surgery.

### 2.1. Radical Prostatectomy

All surgical procedures were performed by a single expert surgeon (AIH), according to the recently described surgical technique [29]. RP was performed via the same suprapubic approach, after the completion of ePLND. Whenever it was technically feasible and oncologically justified, unilateral or bilateral preservation of the neurovascular bundles (NVBs) was implemented. In case of clinically organ-confined PCa, associated with preserved potency prior to the operation, all efforts were done to spare bilaterally the NVBs, as well as the bladder neck. This was rarely possible in case of clinically locally advanced PCa, where wide excision of the NVBs on one or both sides of the prostate was intentionally performed. In any such case, the excision extended to the anterior wall of the rectum, including in the specimen both layers of the Denonvilliers' fascia. The bladder neck was intentionally sacrificed, as well, and a “tennis racket” type bladder neck reconstruction was done after specimen's removal.

 All surgical specimens were fixed in neutral formalin and then processed separately for routine histological haematoxylin-eosin (H&E) and immunohistochemical prostate-specific antigen (PSA) and cytokeratin (CK) examination. Frozen section analysis was rarely performed—only in case of suspicious lymph nodes (LNs) found at surgery, with or without the assistance of a gamma probe and a radioactive counter [4]. A positive histological result from the frozen section analysis did not affect the initial decision to remove the prostate and the seminal vesicles.

### 2.2. Adjuvant Treatment

As an adjunct to surgery, adjuvant hormonal therapy and/or radiotherapy was administered according to the current guidelines and the decision of the institutional multidisciplinary Oncological Committee (Table 2).

 In case of pT3-T4 disease, or positive surgical margins, patients received adjuvant radiotherapy (ART) within the first 3 months after surgery. The external beam radiotherapy was realized in two sessions, by the so-called box technique: (1) large volume irradiation, applied to the prostatic bed and the regional pelvic LNs (1.8–2 Gy daily dose, 46–50 Gy total dose); (2) small volume irradiation, additionally applied to the prostatic bed only, thus achieving a total dose of 60–64 Gy.

 In case of persistent or rising PSA after surgery, or in case of lymph node metastases (LNM) found by the morphologists, patients received permanent adjuvant hormonal therapy (combination of a luteinising hormone-releasing hormone analogue and antiandrogen, to achieve a complete androgen blockage), with an option to switch to intermittent androgen deprivation therapy (ADT) after the first disease-free year with permanent undetectable PSA values.

### 2.3. Group Stratification

 Patients were stratified into two groups, according to the initial PSA values prior to RP: *group A*, comprising 131 men with initial PSA ≤20 ng/mL and *group B*, comprising 74 men with initial PSA >20 ng/mL. The two groups were compared with regard to the functional and oncological outcome after surgery.

### 2.4. Statistical Analysis

 Clinicopathological variables and outcome data were compared across the groups using chi-square and log-rank tests. Univariate analysis, based on the Kaplan-Meier method, and multivariate analysis, based on the Cox's proportional hazards regression model, were performed to determine the significant predictors of outcome among men with PSA >20 ng/mL. Commercially available statistical software packages (SPSS for Windows, v. 16.0, and GraphPad Prism, v. 5.04) were used for the purpose. The endpoints of the study were: the BCR-free survival, the overall survival (OS), and the cancer-specific survival (CSS). The BCR-free patient survival was defined as the percentage of PCa patients with no residual or recurrent disease after RP: serum PSA less than 0.2 ng/mL and no clinical evidence of local recurrence and/or distant metastases. OS was defined as the percentage of PCa patients who had been alive after a particular duration of time. CSS was defined as the percentage of PCa patients who had not died due to PCa at a particular point of time.

## 3. Results

 All cases were followed till July 1st, 2011. The mean followup in the entire series was 50.9 months (±46.5 SD).

 Patients in group B with initial PSA >20 ng/mL had significantly higher clinical stage and biopsy Gleason score, and were more likely to have concomitant EPE, LNI, and positive surgical margins (PSMs) on final pathology, as compared to those in group A (Table 1). Neoadjuvant hormonotherapy and adjuvant treatment modalities (ADT and ART) were more commonly used in group B, as compared to group A (all *P* values <0.05) (Table 2).

**Figure 1:**
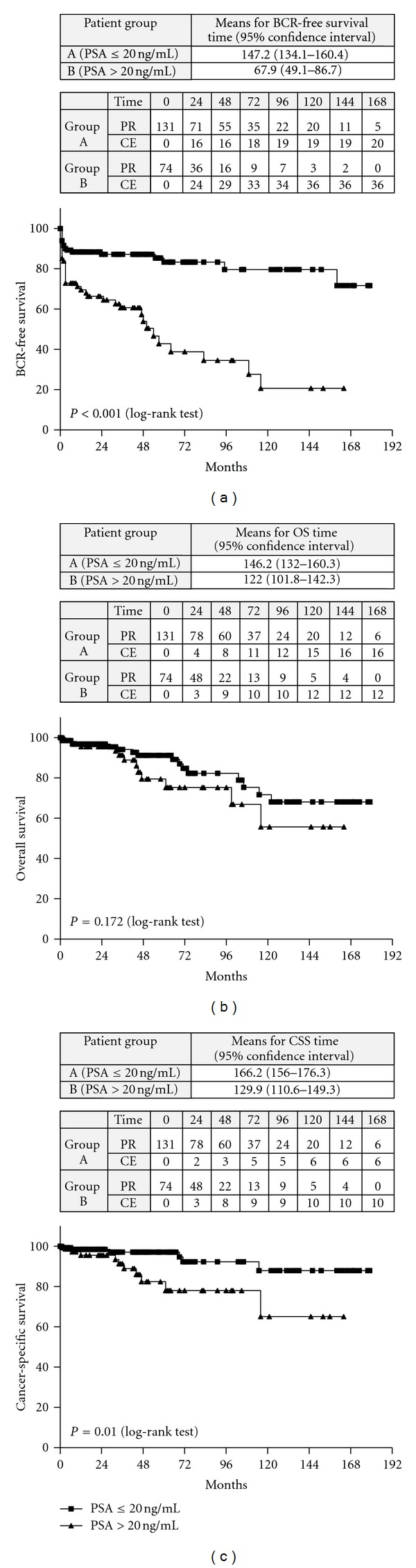
(a) Kaplan-Meyer curves distribution: comparison between patients with PSA ≤20 ng/mL versus patients with PSA >20 ng/mL with regard to BCR-free survival rates. PR: patients at risk; CE: cumulative number of events. (b) Kaplan-Meyer curves distribution: Comparison between patients with PSA ≤20 ng/mL versus patients with PSA >20 ng/mL with regard to OS survival rates. PR: patients at risk; CE: cumulative number of events. (c) Kaplan-Meyer curves distribution: comparison between patients with PSA ≤20 ng/mL versus patients with PSA >20 ng/mL with regard to CSS survival rates. PR: patients at risk; CE: cumulative number of events.

 The Kaplan-Meier survival curves distribution between patients with PSA ≤20 ng/mL and patients with PSA >20 ng/mL is presented in Figure 1. There was a statistically significant difference between curves with regard to the BCR-free survival (Figure 1(a)) (*P* < 0.001) and the CSS rates (Figure 1(c)) (*P* = 0.010). Although lower than in group A, the OS rate of the patients in group B was not significantly altered (Figure 1(b)) (*P* = 0.172).

The Kaplan-Meier estimates of the BCR-free survival, the OS, and the CSS at the 10th year after surgery were 79.6%, 71.7% and 87.9% for patients in group A and 20.7%, 55.7% and 65.0% for patients in group B, respectively (Table 3).

 Using multivariate analysis, the pathological T stage (*P* = 0.009) and the lymph node status (*P* = 0.034) were found to be independent predictors of PSA failure among men with PSA >20 ng/mL (Table 4).

**Figure 2:**
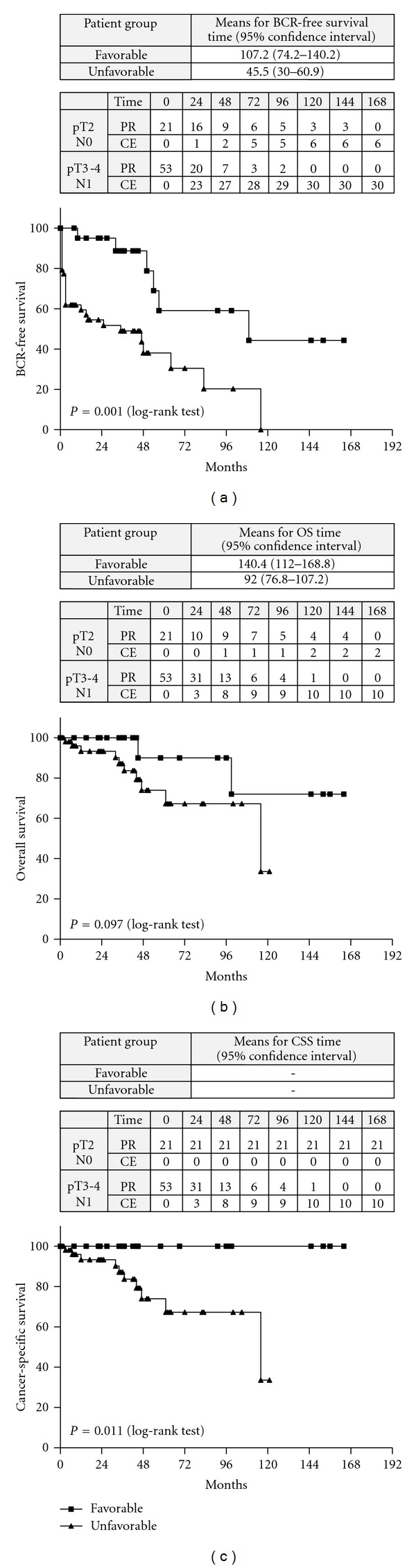
(a) Kaplan-Meyer curves distribution in group B: comparison between patients with favorable versus unfavorable prognostic features with regard to BCR-free survival rates. PR: patients at risk; CE: cumulative number of events. (b) Kaplan-Meyer curves distribution in group B: Comparison between patients with favorable versus unfavorable prognostic features with regard to OS survival rates. PR: patients at risk; CE: cumulative number of events. (C) Kaplan-Meyer curves distribution in group B: comparison between patients with favorable versus unfavorable prognostic features with regard to CSS survival rates. PR: patients at risk; CE: cumulative number of events.

 Patients with favorable combination of these prognostic variables (pT2, N0) had significantly longer BCR-free (*P* = 0.001) (Figure 2(a) and CSS (*P* = 0.011) rates (Figure 2(c)), similar to those of men with initial PSA ≤20 ng/mL. The OS rates were not significantly altered (Figure 2(b)).

The Kaplan-Meier estimates of the BCR-free survival, the OS, and the CSS at the 10th year after surgery in patients with initial PSA >20 ng/mL are shown on Table 5.

There was no statistically significant difference between the patients who received some form of hormonal manipulation prior to surgery, compared to those who did not, with regard to the BCR-free survival (*P* = 0.347), the CSS (*P* = 0.317), and the OS (*P* = 0.091) rates.

**Figure 3:**
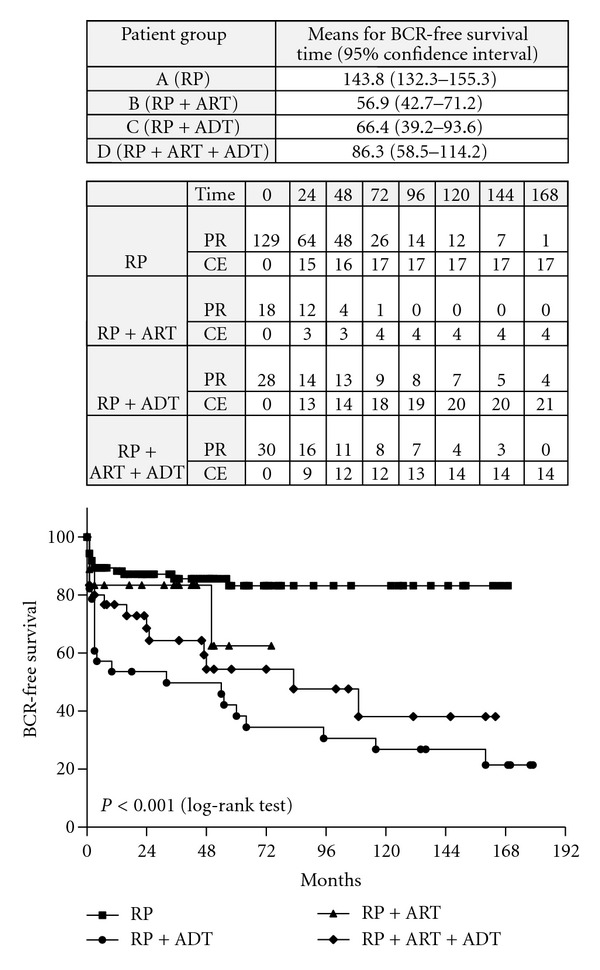
Kaplan-Meyer curves distribution: comparison between patients treated by RP only versus RP plus ART versus RP plus ADT versus RP plus ART plus ADT with regard to BCR-free survival rates. PR: patients at risk; CE: cumulative number of events.

 The univariate analysis, based on the Kaplan-Meier method, showed a statistically significant difference between the four treatment groups (patients treated by RP only versus RP plus ART versus RP plus ADT versus RP plus ART plus ADT) with regard to the BCR-free survival rate (*P* < 0.001, log-rank test) (Figure 3). The Kaplan-Meier estimates of the 10-year BCR-free survival were 83.6%, 62.5%, 26.8% and 38.1% for patients, who were treated by RP only, by RP plus ART, by RP plus ADT, and by combination of all treatment modalities (RP, ART and ADT), respectively.

The results of the Kaplan-Meier survival analysis in the different treatment groups are shown on Table 6. In all groups of patients there was no statistically significant difference between men with initial PSA ≤20 ng/mL versus those with PSA >20 ng/mL with regard to OS and CSS rates (all *P* values >0.05, log-rank test).

## 4. Discussion

 Although PSA is an established prognostic variable, its high values to some extent limit its predictive accuracy. These high levels are often due to a large prostate weight, or to a large volume of a tumor, being otherwise localized within the prostate. For that reason some authors suggest that a high PSA value is an insufficient indicator of a proper treatment [16, 22].

 Anyway, PCa patients with initial serum PSA values above 20 ng/mL are generally considered as a “high-risk group”, suggesting a poor oncological outcome [7, 9, 21]. Therefore, they are often rejected as potential candidates for definitive local treatment.

Some of these cases, however, respond favorably to radical surgery. Nguyen et al. [30] recently reported that more than 50% of their PCa patients with initial PSA values above 20 ng/mL remained with undetectable PSA values during the first 5 years after RP. This result is in agreement with other patient series, where the 5-year biochemical recurrence-free (BCR-free) survival is within the range between 48% and 65% [5, 12, 14, 18]. In the majority of these cases favorable results had been achieved by RP, used as monotherapy, without the application of adjuvant treatment strategies [14, 18, 30].

These results support the fact that RP might be considered as a viable treatment option in selected high-risk patients [12, 16, 20, 21, 31].

 In many cases, however, locally advanced disease or recurrence after RP had been found, necessitating second-line therapy (ADT and/or ART). Therefore, all patients with PSA values above 20 ng/mL should initially be warned that surgery might not be sufficient to control PCa, and adjuvant treatment modalities might be used at a later time [32].

 In the absence of large scale, multicenter, randomised prospective trials, comparing early versus deferred adjuvant treatments, it is difficult to decide when to start adjuvant therapy in this particular patient subset. In our study ADT was applied in 39.2%; ART in 36.5%, and combined adjuvant therapy (ADT plus ART)—in 23.0% of the cases. Our current treatment strategy is to use these two methods only in case of clear, distinct indications: locally advanced disease (EPE, SVI, PSM, and/or LNI), or biochemical recurrence after RP (raise in PSA above the cut-point value of 0.2 ng/mL).

 There is obviously a need for better identification of the subgroup of patients with initial serum PSA >20 ng/mL, who are more likely to benefit from RP.

 Briganti et al. [20] reported that roughly 40% of patients with high-risk PCa had specimen-confined disease at final pathology—namely, pT2-pT3a, node negative PCa with negative surgical margins. These patients showed excellent outcomes in the long term, thus representing the ideal candidates for RP as a primary treatment. The authors suggested a nomogram based on routinely available clinical parameters (age and PSA level at surgery, Gleason score at biopsy, and clinical stage) to better identify the subset of high-risk PCa patients who might have favorable pathologic outcomes when surgically treated.

 Our results corroborate these findings. The pathological tumor stage and the LN status were found to be the only independent prognostic variables to predict the BCR-free patient survival among men with PSA >20 ng/mL at the time of RP. Patients with favorable combination of these prognostic variables, that is, patients with specimen-confined disease (pT2, N0), had significantly longer BCR-free (*P* = 0.001) and CSS (*P* = 0.011) rates, similar to those of men with initial PSA ≤20 ng/mL.

 Recently, it has been shown that multiparameter MRI of the prostate can detect initial EPE, and even distinguish benign from neoplastic tissue with a promising specificity [33, 34]. The current improvements of MRI and other imaging modalities used for diagnosis and staging will lead to a more accurate definition of the tumor stage, which is particularly important in patients with PSA values above 20 ng/mL.

 Our study, however, has a few limitations that have to be taken into consideration.

 Firstly, the total number of patients, comprising the study, was quite low (*n* = 205). Patient number was even lower within each subgroup analyzed. For that reason, the KM curves and all other results achieved should be interpreted with caution.

Secondly, too many patients (roughly one third of the entire series) had some kind of hormonal manipulation prior to surgery (neoadjuvant hormonotherapy and/or bilateral orchiectomy). The decision to do that had been taken by the urologists at the primary urological institution, probably because of the adverse clinical and pathological characteristics of the patients and their tumors. The majority of these cases belong to our early series, when neoadjuvant hormonal treatment was a common practice. This strategy continues to be used, even nowadays, in some European centers [19]. Nevertheless, when later reassessed in our institution, which functions as a tertiary referral center for the North-Eastern part of the country, all these 71 patients were found eligible for surgical treatment and subjected to radical prostatectomy.

 One might think that this manipulation would have an impact on patient outcome. In a profound review and meta-analysis, Shelley et al. [35] studied the role of neoadjuvant hormonal therapy and RP. The authors reported that this type of treatment does substantially improve local pathological variables, such as organ-confined rates, pathological downstaging, PSM, and rate of LNI, but does not provide significant BCR-free, CSS and OS advantages over RP alone. Therefore, neoadjuvant hormonal therapy is no longer recommended to patients who will be subjected to radical surgical treatment. Our study also confirmed that this type of treatment had no impact on patient survival.

 Another limitation of our study is that the majority of our patients received some form of adjuvant treatment (ART and/or ADT) after radical surgery. Accumulated evidence in the literature shows that patient outcomes are largely altered by the use of adjuvant treatment options. In order to assess this issue we divided our patients into four groups with respect to the mode of treatment applied: RP only, RP plus ART, RP plus ADT, and RP plus combination from ART and ADT. We found that there was statistically significant difference (*P* < 0.001) between KM curves when the BCR-free patient survival was used as a study end-point. Interestingly, the highest BCR-free survival was found among patients left without any adjuvant treatment after surgery. This ostensible paradox could be explained by the fact that this patient group usually comprises patients with favorable pathological characteristics which do not require the application of adjuvant treatment modalities, like ART and/or ADT. It was also interesting to note that there were no statistically significant differences between group A and group B when the CSS and OS were used as study end-points. Although the patient numbers in each of the previously mentioned four treatment groups are low and for that reason cannot lead to definite conclusions, this result means that RP, either alone or as part of a multimodal treatment, is a viable treatment option even in patients with PSA values above 20 ng/mL at the time of radical surgery.

In spite of all these limitations, our study provides some evidence that patients with PSA values above 20 ng/mL should not be uniformly considered as a high-risk group. Among them, there are many patients with favorable pathologic characteristics, who might also benefit from radical surgical treatment, applied either alone, or as part of a multimodal treatment approach.

 As there is paucity in the current literature regarding this specific matter [13, 14, 18], other studies are sorely needed to confirm our results.

## 5. Conclusions

High initial PSA values do not uniformly indicate poor prognosis after radical prostatectomy. This operation can still be considered as a viable therapeutic option, even in PCa patients with initial serum PSA values above 20 ng/mL. Patients, who might benefit the most from complete surgical excision, are those with organ confined prostate cancer and negative lymph nodes.

## Figures and Tables

**Table 1:** Patient characteristics and pathological parameters.

Parameter	Group A (*n* = 131)	Group B (*n* = 74)	*P* value
Patient age (years) ± SD	65.7 ± 6.1	65.4 ± 7.7	0.760
Mean PSA (ng/mL) ± SD	9.4 ± 5.4	64.9 ± 123.9	<0.001
Clinical stage (*n*/%/)^∗^			
cT1	32 (24.4%)	3 (4.1%)	<0.001
cT2	91 (69.5%)	43 (58.1%)	0.101
cT3-T4	8 (6.1%)	28 (37.8%)	<0.001
Gleason score (*n*/%/)			
<7	52 (39.7%)	13 (17.6%)	0.001
=7	55 (42.0%)	28 (37.8%)	0.557
>7	24 (18.3%)	33 (44.6%)	<0.001
Pathological stage (*n*/%/)^∗ ^			
pT2	89 (67.9%)	24 (32.4%)	<0.001
pT3	37 (28.2%)	42 (56.8%)	<0.001
pT4	5 (3.8%)	8 (10.8%)	0.049
Extracapsular extension (*n*/%/)	42 (32.1%)	50 (67.6%)	<0.001
Seminal vesicles invasion (*n*/%/)	35 (26.7%)	45 (60.8%)	<0.001
Lymph node involvement (*n*/%/)	19 (14.5%)	35 (47.3%)	<0.001
Positive surgical margins (*n*/%/)	20 (15.3%)	31 (41.9%)	<0.001

^∗^Based on TNM classification, v. 2009.

**Table 2:** Neoadjuvant and adjuvant treatment modalities.

Parameter	Group A (*n* = 131)	Group B (*n* = 74)	*P* value
Neoadjuvant hormonal therapy (*n*/%/)	38 (29.0%)	33 (44.6%)	0.025
Adjuvant radiotherapy (ART) (*n*/%/)	21 (16.0%)	27 (36.5%)	0.001
Adjuvant hormonal therapy (ADT) (*n*/%/)	29 (22.1%)	29 (39.2%)	0.010
Adjuvant combined (ART & ADT) therapy (*n*/%/)	13 (9.9%)	17 (23.0%)	0.012

**Table 3:** Oncological outcome at the 10th year after surgery.

Patient group	BCR-free survival		Overall survival		Cancer-specific survival	
	% Censored cases	KM estimates (10th year)	% Censored cases	KM estimates (10th year)	% Censored cases	KM estimates (10th year)
A (PSA ≤20 ng/mL)	84.7%	79.6%	87.8%	71.7%	95.4%	87.9%
B (PSA >20 ng/mL)	51.4%	20.7%	83.8%	55.7%	86.5%	65.0%
*P* value	**<** **0.001**		**0.172**		**0.010**	

**Table 4:** Univariate and multivariate analysis of pathologic variables.

Parameter	Univariate analysis	Multivariate analysis	
	*P *value	*P *value	HR^∗^ (95% CI^∗∗^)
Age (years)	0.164	0.506	—
Initial PSA (ng/mL)	0.042	0.116	—
cT (cT1 versus cT2 versus cT3)	0.003	0.806	—
Gleason score (<7 versus 7 versus >7)	0.002	0.065	—
pT (pT2 versus pT3 versus pT4)	**<0.001**	**0.009 **	**3.515 (1.882–6.565) **
Seminal vesicle invasion (yes versus no)	0.006	0.932	—
Surgical margins (neg. versus pos.)	0.003	0.084	—
Lymph node status (N0 versus N1)	**<0.001**	**0.034 **	**1.002 (1.000–1.003) **
Adjuvant radiotherapy (yes versus no)	0.968	0.506	—
Adjuvant hormonal therapy (yes versus no)	0.023	0.105	—

^∗^HR: hazard ratio; ^∗∗^CI: confidence interval.

**Table 5:** Oncological outcome at the 10th year after surgery in patients with favorable combination of prognostic variables (pT2, N0) versus patients with unfavorable prognostic variables (pT3-4 and/or N1).

Patient group	BCR-free survival		Overall survival		Cancer-specific survival	
	% Censored cases	KM estimates (10th year)	% Censored cases	KM estimates (10th year)	% Censored cases	KM estimates (10th year)
Favorable (pT2, N0)	71.4%	44.3%	90.5%	72.0%	100.0%	100.0%
Unfavorable (pT3-4 and/or N1)	43.4%	0%	81.1%	33.6%	81.1%	33.6%
*P* value	** 0.001**		**0.097**		**0.011**	

**Table 6:** Kaplan-Meier survival analysis, log-rank test: comparison between patients with initial PSA ≤20 ng/mL versus patients with PSA ≤20 ng/mL in four patient groups (patients treated by RP only versus RP plus ART versus RP plus ADT versus RP plus ART plus ADT) with regard to BCR-free, OS, and CSS rates.

Patient group	*P *value		
	BCR-free survival	Overall survival	Cancer-specific survival
RP	0.002	0.501	0.155
RP + ART	0.034	0.315	0.101
RP + ADT	0.008	0.312	0.206
RP + ART + ADT	0.221	0.238	0.238
